# Transcriptomic evidence for tumor‐specific beneficial or adverse effects of TGFβ pathway inhibition on the prognosis of patients with liver cancer

**DOI:** 10.1002/2211-5463.13647

**Published:** 2023-05-31

**Authors:** Matthis Desoteux, Betty Maillot, Kevin Bévant, Tanguy Ferlier, Raffaële Leroux, Gaëlle Angenard, Corentin Louis, Laurent Sulpice, Karim Boudjema, Cédric Coulouarn

**Affiliations:** ^1^ Inserm, UMR_S 1242, OSS (Oncogenesis Stress Signaling) Centre de Lutte contre le Cancer Eugène Marquis, Univ Rennes France; ^2^ Department of Hepatobiliary and Digestive Surgery Rennes University Hospital France; ^3^ Inserm, Inrae, UMR_S 1317 NuMeCan (Nutrition, Metabolisms and Cancer), Univ Rennes France; ^4^ Clinical Investigation Center 1414 CHU de Rennes France

**Keywords:** galunisertib, gene signature, hepatocellular carcinoma, survival, TGFβ

## Abstract

Therapeutic targeting of the transforming growth factor beta (TGFβ) pathway in cancer represents a clinical challenge since TGFβ exhibits either tumor suppressive or tumor promoting properties, depending on the tumor stage. Thus, treatment with galunisertib, a small molecule inhibitor of TGFβ receptor type 1, demonstrated clinical benefits only in subsets of patients. Due to the functional duality of TGFβ in cancer, one can hypothesize that inhibiting this pathway could result in beneficial or adverse effects depending on tumor subtypes. Here, we report distinct gene expression signatures in response to galunisertib in PLC/PRF/5 and SNU‐449, two cell lines that recapitulate human hepatocellular carcinoma (HCC) with good and poor prognosis, respectively. More importantly, integrative transcriptomics using independent cohorts of patients with HCC demonstrates that galunisertib‐induced transcriptional reprogramming in SNU‐449 is associated with human HCC with a better clinical outcome (i.e., increased overall survival), while galunisertib‐induced transcriptional reprogramming in PLC/PRF/5 is associated with human HCC with a worse clinical outcome (i.e., reduced overall survival), demonstrating that galunisertib could indeed be beneficial or detrimental depending on HCC subtypes. Collectively, our study highlights the importance of patient selection to demonstrate a clinical benefit of TGFβ pathway inhibition and identifies Serpin Family F Member 2 (SERPINF2) as a putative companion biomarker for galunisertib in HCC.

## Graphical abstract

By using clinically relevant human hepatocellular carcinoma (HCC) cell lines and integrative transcriptomics, this study provides evidence that galunisertib‐induced transcriptional reprogramming is associated with beneficial or adverse effects depending on tumor subtypes. The study demonstrates that transforming growth factor beta (TGFβ) pathway inhibition should be restricted to HCC subtypes with a mesenchymal phenotype in which the tumor suppressive arm of TGFβ is lost.

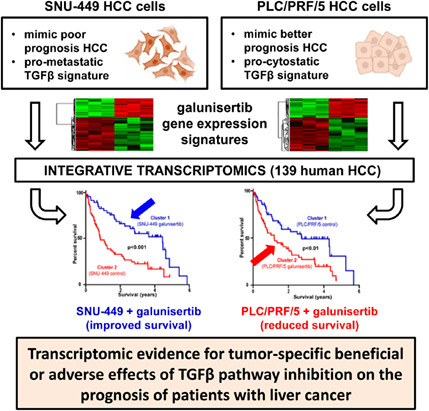

**Abbreviations**
AFP: alpha‐fetoprotein
ATCC: American Type Culture Collection
BCLC: Barcelona Clinic Liver Cancer
CDH1: E‐cadherin
EMT: epithelial‐to‐mesenchymal transition
GSEA: gene set enrichment analysis
HCC: hepatocellular carcinoma
ICI: immune checkpoint inhibitors
MSigDB: molecular signatures database
qRT‐PCR: quantitative real‐time polymerase chain reaction
SERPINF2: serpin family F member 2
TCGA: The Cancer Genome Atlas
TGFβ: transforming growth factor beta
TNM: tumor node metastasis
VIM: vimentin

Hepatocellular carcinoma (HCC) is a deadly cancer worldwide due to limited therapeutic options [1]. Surgery remains the best strategy for curative intent when performed at an early stage [2]. However, most HCC are diagnosed at an advanced stage and patients are then evaluated for systemic treatments [2, 3]. Although phase III clinical trials reported positive results for antiangiogenic drugs, alone or combined with immune checkpoint inhibitors (ICI) [2, 4], extending the therapeutic options to reduce vascular invasion and metastatic progression still represents an important need. Targeting the transforming growth factor beta (TGFβ) pathway has been proposed as a promising strategy in HCC [5]. Indeed, as a potent inducer of epithelial‐to‐mesenchymal transition (EMT), TGFβ contributes to metastasis [6]. TGFβ is also a potent immunosuppressive cytokine, which has been associated with cold tumors and resistance to ICI [7]. Thus, several molecules have been developed to inhibit the TGFβ pathway, including galunisertib (LY2157299), a selective ATP‐mimetic inhibitor of type 1 TGFβ receptor [5]. In preclinical models, galunisertib enhanced sorafenib‐induced apoptosis and reduced stemness‐related functions of invasive HCC cells [8, 9, 10]. Phase 1 studies demonstrated a favorable toxicity and pharmacokinetic profiles [11, 12]. However, phase 2 studies demonstrated a clinical benefit only in subsets of patients, a feature that could be explained by the functional duality of TGFβ [12, 13]. Actually, if contributing to tumor progression and resistance to therapies, TGFβ can also exert cytostatic actions limiting tumor growth in some HCC cases, for which its inhibition could be at best ineffective but at worse detrimental by promoting tumor progression. In this study, by using clinically relevant human HCC cell lines and integrative transcriptomics, we provide evidence that a galunisertib‐induced transcriptional reprogramming is associated with beneficial or adverse effects depending on tumor subtypes. The study demonstrates that TGFβ pathway inhibition should be restricted to HCC subtypes with a mesenchymal phenotype in which the tumor suppressive arm of TGFβ is lost. The study also identifies Serpin Family F Member 2 (SERPINF2) as a putative companion biomarker for galunisertib to identify the patients who may benefit from galunisertib treatment.

## Materials and methods

### Cell culture

PLC/PRF/5 (CRL‐8024) and SNU‐449 (CRL‐2234) HCC cell lines were purchased from the American Type Culture Collection (ATCC, Manassas, VA, USA, www.lgcstandards‐atcc.org) and cultured as previously described [14, 15]. ATCC performed cell line authentication by Short Tandem Repeat DNA profiling. No alteration (i.e., mutation, fusion, or translocation) in *SMAD2‐4* and *CTNNB1* genes was observed in PLC/PRF/5 and SNU‐449 cell lines (https://depmap.org/portal/). Cells were treated for 16 h with 10 μm galunisertib, also known as LY2157299 (Sigma‐Aldrich, St. Louis, MO, USA), alone or in combination with 1 ng·mL^−1^ recombinant human TGFβ1 (R&D Systems, Minneapolis, MN, USA) after overnight serum starvation [15]. All cell cultures were conducted at 37 °C in a 5% CO_2_ atmosphere. Independent culture experiments were carried out in triplicate. RNA was extracted after 16 h of treatment and submitted to gene expression profiling.

### Gene expression profiling

Total RNA was purified from cells at 80% confluency with an RNeasy kit (Qiagen, Courtaboeuf, France). Genome‐wide expression profiling was conducted using low‐input Quick Amp labeling kits and human SurePrint G3 pangenomic (60K probes) microarrays (Agilent Technologies, Les Ulis, France), as previously described [14]. Gene expression data were processed using feature extraction and genespring softwares (Agilent Technologies) and further analyzed using R‐based BRB‐ArrayTools, as described [16]. Clustering analysis was made using cluster 3.0 and treeview 1.6 using uncentered correlation and average linkage options. Gene expression datasets generated in this study are openly available in Gene Expression Omnibus (accession number GSE211806).

### Data mining and integrative transcriptomics

Gene annotation was based on Gene Ontology and enrichment for specific biologic functions or canonical pathways was evaluated using funcassociate 2.0 program [17]. Gene set enrichment analysis (GSEA) was conducted by using the Java tool developed at the Broad Institute (Cambridge, MA, USA), as previously described [18]. Unsupervised GSEA was done with the whole C2 collection of curated gene sets from the molecular signatures database (MSigDB). Integration of transcriptomic data was conducted using publicly available HCC datasets (GSE1898, GSE4024, and GSE14520), as previously described [15, 16, 19]. In total, two independent cohorts of HCC were analyzed. The first cohort (GSE1898 and GSE4024) included 139 HCC [16, 20, 21, 22] and the second cohort (GSE14520) included 225 HCC [23]. Survival analysis was also performed using The Cancer Genome Atlas (TCGA) datasets (https://portal.gdc.cancer.gov/projects/TCGA‐LIHC).

### Quantitative real‐time PCR

Expression of relevant genes was measured by quantitative real‐time PCR (qRT‐PCR) using SYBR Green (Applied Biosystems, Carlsbad, CA, USA), as previously described [15]. Quantitative analysis of PCR data was conducted with the 2^–∆∆Ct^ method. Melting curves analysis was used to validate the specificity of PCR products.

### Statistical analysis

Gene expression profiling was made using RNA extracted from three independent culture experiments. Single gene expression data are presented as the mean ± standard deviation. Statistical analyses were performed using r‐3.5.1 and graphpad prism 7.0, Boston, MA, USA. For microarray data, differentially expressed genes were identified by a two‐sample univariate *t*‐test and a random variance model [24]. For group comparison of quantitative variables, *t*‐testing was applied. Categorical data were analyzed by chi‐squared testing. The cumulative survival rate was estimated by the Kaplan–Meier method, and the survival curves were compared with the log‐rank test. *P*‐values below 0.05 were considered as significant statistical differences.

## Results and Discussion

### PLC/PRF/5 and SNU‐449 reproduce human HCC with different prognosis

**Fig. 1:**
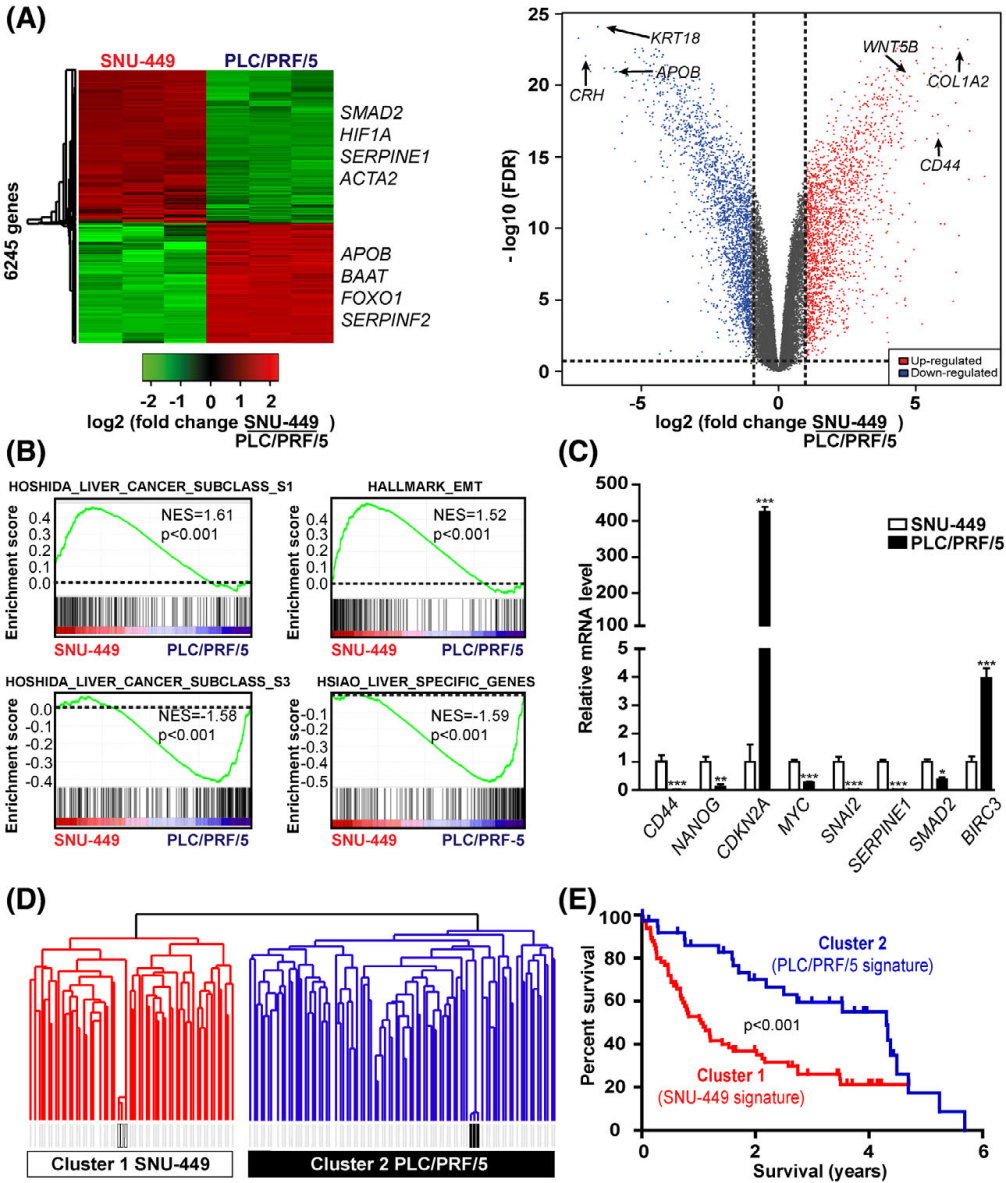
PLC/PRF/5 and SNU‐449 cells mimic two groups of human HCC with a variable prognosis. (A) Hierarchical clustering and volcano plot of genes differentially expressed between PLC/PRF/5 and SNU‐449 (dashed lines: fold‐change FC > 2; *P* < 0.001; *n* = 3 independent experiments). The complete list of genes is provided in Table S1. (B) GSEA using the gene expression profiles of SNU‐449 and PLC/PRF/5 cells and signatures associated with liver cancer (NES, normalized enrichment score provided by GSEA). (C) Expression of key genes representative of TGFβ‐related functions. Data are presented as bar plots with mean ± SD and were compared using the Student's *t*‐test, *P < 0.05, **P < 0.01, and ***P < 0.001 (*n* = 3 independent experiments). (D) Dendrogram overview of PLC/PRF/5 and SNU‐449 gene expression profiles integrated with those of 139 human HCC cases (GSE1898 and GSE4024). Clustering analysis identified two major HCC clusters associated with SNU‐449 (cluster 1) and PLC/PRF/5 (cluster 2) profiles. (E) Kaplan–Meier plot and log‐rank statistics of HCC included in clusters 1 and 2.

Hepatocellular carcinoma cell lines constitute relevant models to investigate signaling pathways altered in liver cancer and to test new drug candidates [14, 25, 26, 27, 28]. However, to what extent these cell lines recapitulate the biology of human HCC tumors has been partly addressed [29]. Here, we evaluated the clinical relevance of PLC/PRF/5 and SNU‐449 cell lines, which were representative of two clusters differing notably in the expression of genes associated with metastasis in a panel of 19 HCC cell lines [16]. In addition, from this panel, we previously demonstrated by integrative transcriptomics that PLC/PRF/5 and SNU‐449 cell lines recapitulated human HCC with tumor‐suppressive and tumor‐promoting features of the TGFβ pathway, respectively [16]. Accordingly, PLC/PRF/5 and SNU‐449 differed in the expression of epithelial and mesenchymal markers, SNU‐449 exhibiting a mesenchymal‐like phenotype and a higher secretion of TGFβ (Fig. S1), in agreement with previously published data [15, 30]. It was also shown that a mesenchymal‐like phenotype in HCC cell lines correlated with autocrine stimulation of the TGFβ pathway and resistance to TGFβ‐induced suppressor effects [30]. Not surprisingly, numerous genes were differentially expressed between PLC/PRF/5 and SNU‐449 cells (Fig. 1A; Table S1). A significant enrichment of signatures associated with a previously described molecular classification of human HCC [31] was highlighted. Thus, the HOSHIDA_LIVER_CANCER SUBCLASS_S1 and SUBCLASS_S3 signatures were enriched in the gene expression profiles of SNU‐449 and PLC/PRF/5 cells, respectively (Fig. 1B). The S1 subtype reflected an aberrant activation of the WNT pathway through TGFβ, while the S3 subtype was associated with a preserved hepatocyte differentiation [31]. Supporting this observation, the HALLMARK_EMT [32] and the HSIAO_LIVER_SPECIFIC_GENES [33] signatures were enriched in the gene expression profiles of SNU‐449 and PLC/PRF/5 cells, respectively (Fig. 1B). In agreement with a more differentiated phenotype, key genes associated with liver metabolism (e.g., *APOB*) were more expressed in PLC/PRF/5 than in SNU‐449 cell line. Conversely, genes associated with cancer stem cells (e.g., *CD44*), TGFβ pathway (e.g., *SERPINE1*), and EMT (e.g., *SNAI2*) were expressed at a higher level in SNU‐449 than in PLC/PRF/5 cell line (Fig. 1C), recapitulating previous reports made at transcriptomic and proteomic levels [27, 29]. Supporting a higher proliferative potential of SNU‐449 cells, cyclin‐dependent kinase inhibitor 2A (*CDKN2A*) and *BIRC3*, encoding an inhibitor of apoptosis, were underexpressed while *MYC* was overexpressed (Fig. 1C). Next, we integrated the signature made of genes differentially expressed between SNU‐449 and PLC/PRF/5 cell lines with the gene expression profiles of 139 HCC cases with clinical annotations [16, 21, 22]. Clustering analysis of the integrated datasets identified two groups of human HCC associated with either SNU‐449 (cluster 1) or PLC/PRF/5 (cluster 2) cell line (Fig. 1D). Interestingly, patients with HCC that recapitulate the gene expression signature of the PLC/PRF/5 cell line (cluster 2) had a better survival (Fig. 1E). Altogether, gene expression profiling at basal level and integrative transcriptomics demonstrated that PLC/PRF/5 and SNU‐449 cell lines are relevant models to study human HCC with different prognosis.

### Specific galunisertib signatures in PLC/PRF/5 and SNU‐449 cells

**Fig. 2:**
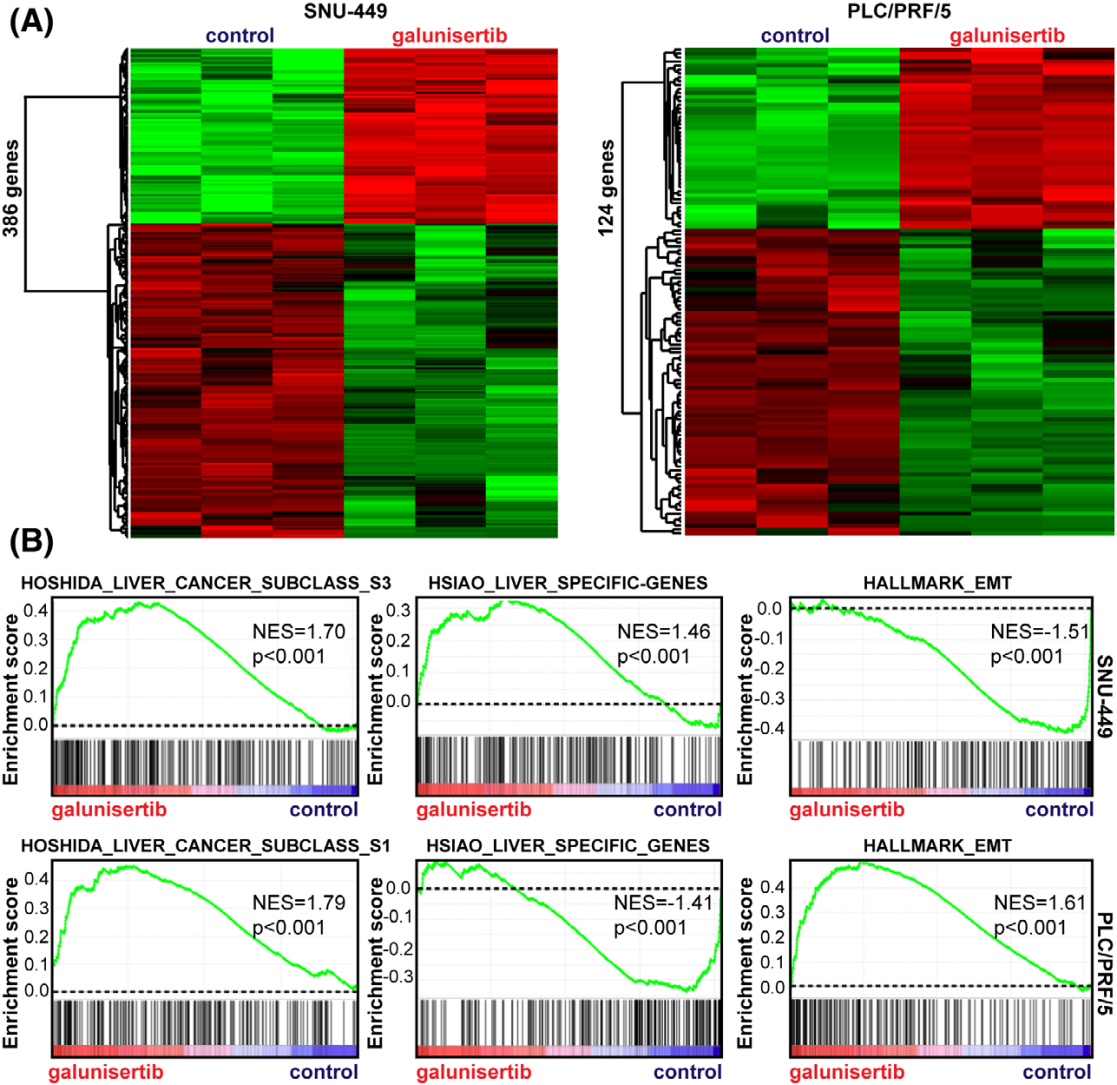
Galunisertib signatures in PLC/PRF/5 and SNU‐449 cells. (A) Hierarchical clustering of genes up‐ and down‐regulated in SNU‐449 (left panel) and PLC/PRF/5 (right panel) after a galunisertib treatment (10 μm, 16 h) (FC > 2; *P* < 0.001). (B) GSEA using the gene expression profiles of SNU‐449 (upper panel) and PLC/PRF/5 (lower panel) after a treatment with galunisertib vs control. GSEA highlighted a cell‐specific enrichment of signatures associated with the molecular classification of human HCC (e.g., HOSHIDA_LIVER_CANCER_SUBCLASS_S1 and SUBCLASS_S3 [31]), hepatocyte differentiation (HSIAO_LIVER_SPECIFIC_GENES [33]) and EMT (HALLMARK_EMT [32]).

Transforming growth factor beta plays a key role in liver carcinogenesis but targeting this pathway is challenging due to its functional duality [34]. Supporting this hypothesis, we previously reported signatures associated with the tumor‐suppressive and tumor‐promoting properties of TGFβ in human HCC with good and bad prognosis, respectively [16]. As we previously reported that PLC/PRF/5 and SNU‐449 cell lines were associated with this functional duality [16], we hypothesized that they may constitute relevant models to infer the impact of inhibiting the TGFβ pathway in specific human HCC. Further supporting this hypothesis, the data above demonstrated that the SNU‐449 cell line is also associated with human HCC from the S1 subtype in which the tumor promoting arm of TGFβ is activated (Fig. 1B). Thus, we established gene expression signatures of TGFβ pathway inhibition by treating PLC/PRF/5 and SNU‐449 cell lines with galunisertib [35]. Galunisertib treatment resulted in the modulation of 386 nonredundant and well‐annotated genes in SNU‐449 cell line (139 upregulated and 247 downregulated versus control) and 124 genes in PLC/PRF/5 cell line (26 upregulated and 78 downregulated versus control) (Fig. 2A; Tables S2 and S3). Only five genes were commonly deregulated in the two cell lines, demonstrating that galunisertib induces cell‐specific changes, similar to TGFβ [36]. Importantly, GSEA suggested that galunisertib could switch the gene expression profile of SNU‐449 cell line toward a more favorable prognosis. Indeed, a positive enrichment of gene signatures associated with a better HCC differentiation (e.g., HOSHIDA_LIVER_CANCER_SUBCLASS_S3 and HSIAO_LIVER_SPECIFIC_GENES), as described above (Fig. 1B), was observed in the gene expression profile of SNU‐449 cells treated with galunisertib, as compared to untreated control cells (Fig. 2B). Conversely, a negative enrichment of the HALLMARK_EMT signature was observed, suggesting that galunisertib may switch the EMT signature toward a MET signature (Fig. 2B). Accordingly, an increased expression of the epithelial marker E‐cadherin (*CDH1*) was observed in SNU‐449 cells treated with galunisertib (Table S2). These results are in agreement with previous data reporting that galunisertib reduces the stemness‐gene profile of invasive HCC cells (including the SNU‐449 cell line) and induces the acquisition of a more epithelial‐like morphology [8, 30]. Galunisertib was also shown to reduce the migration, clonogenicity and the spheroid formation ability of HCC invasive cells [8, 30, 37]. Interestingly, an opposite picture was highlighted for the PLC/PRF/5 cell line since a positive enrichment of unfavorable signatures was observed in cells treated with galunisertib, including the HOSHIDA_LIVER_CANCER SUBCLASS_S1 and the HALLMARK_EMT signatures (Fig. 2B). In addition, a negative enrichment of the HSIAO_LIVER_SPECIFIC_GENES signature was observed, suggesting that galunisertib may switch the PLC/PRF/5 cell line toward a less differentiated phenotype (Fig. 2B). Mechanistically, we hypothesize that liver‐enriched transcription factors involved in maintaining a well‐differentiated hepatocyte phenotype are repressed by galunisertib in the PLC/PRF/5 cell line and induced in the SNU‐449 cell line. Supporting this hypothesis, galunisertib was shown to reduce the expression of *HNF1B*, *HNF4A*, and *ONECUT1* (aka *HNF6A*) involved in maintaining a well‐differentiated hepatocyte‐like phenotype in the PLC/PRF/5, while it induced the expression of *HNF1A*, *FOXA2* (aka *HNF3B*), and *ONECUT1* in SNU‐449 (Fig. S2). Collectively, the results highlighted a differential galunisertib‐induced transcriptional reprogramming in PLC/PRF/5 and SNU‐449 cells possibly associated with different prognosis in human HCC. It is important to note that galunisertib is a small selective kinase inhibitor for TGFβ, activin and nodal type I serine/threonine kinase receptors. Thus, we cannot exclude that part of the observed signatures is also connected to the inhibition of activin and nodal pathways, which have been also associated with HCC. In addition, TGFβ can signal via SMAD and non‐SMAD signaling pathways. Such non‐SMAD signaling pathways like TGFβ/PI3K/AKT or TGFβ/NFKB are thought to play a role in aggressive cancers possibly in a type I receptor kinase‐independent manner (and thus are not blocked by galunisertib). Accordingly, enrichments of PI3K and NFKB signatures were observed upon TGFβ exposure in PLC/PRF/5 and SNU‐449 cell lines. However, upon galunisertib exposure, these enrichments were not statistically significant anymore suggesting that they were dependent on type I receptor kinase (Fig. S3). Thus, one can assume that in our experimental conditions, most of the observed effects on gene expression upon galunisertib exposure are related to the SMAD canonical signaling.

### Galunisertib‐ and TGFβ‐associated signatures in PLC/PRF/5 and SNU‐449 cells identified human HCC with different prognosis

**Fig. 3:**
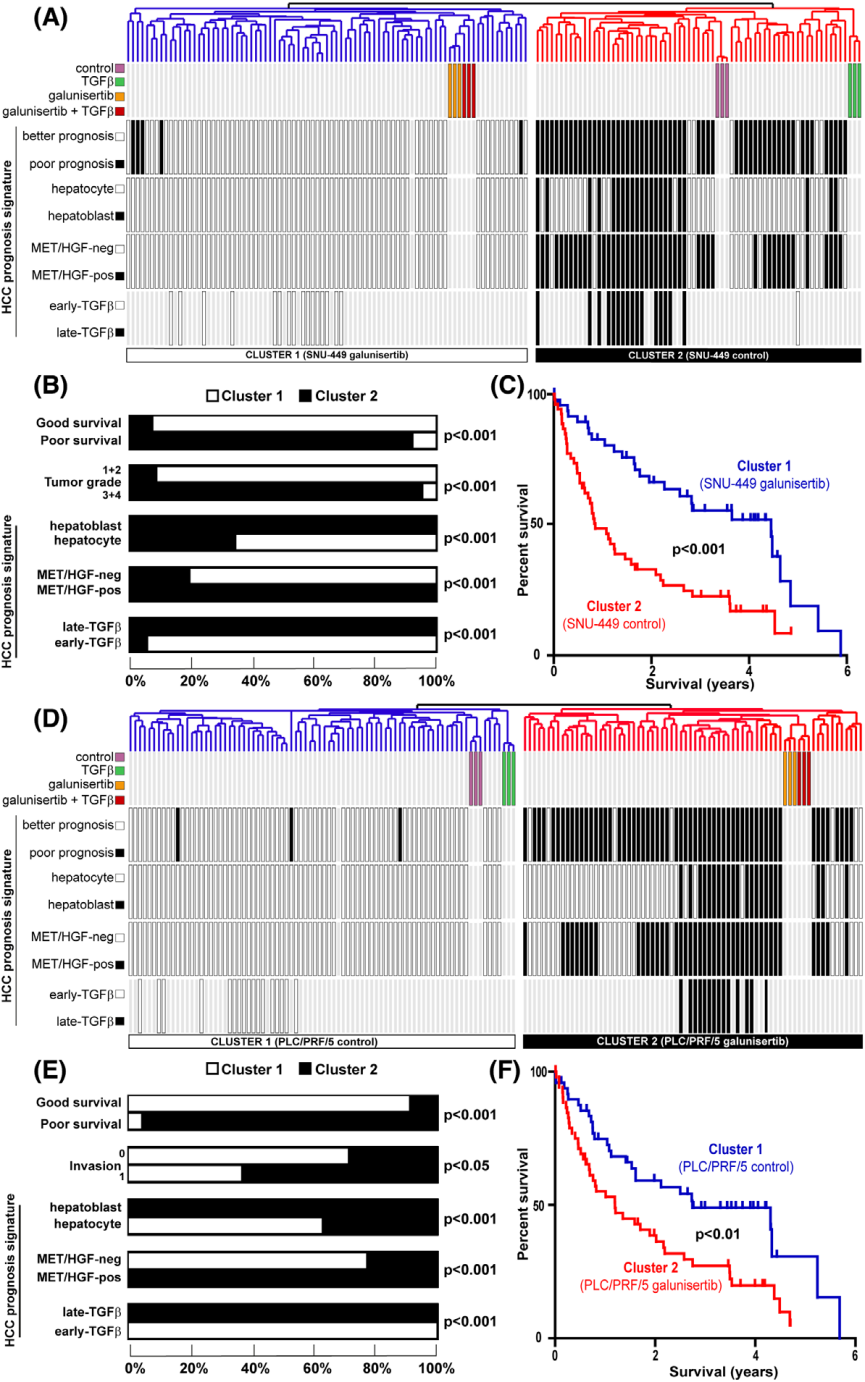
Clinical relevance of galunisertib‐ and TGFβ‐associated signatures derived from SNU‐449 and PLC/PRF/5 cells. (A) Dendrogram overview of *in vitro* experiments performed in SNU‐449 cells integrated with 139 cases of human HCC. Clustering analysis was based on the expression of genes differentially expressed between the four conditions (i.e., SNU‐449 cells treated with TGFβ and galunisertib, alone or in combination, versus untreated control cells). Two main clusters were identified. Cluster 1 included cells treated with galunisertib (in presence and absence of TGFβ co‐treatment) and cluster 2 included untreated control cells and cells treated with TGFβ. Distribution of human HCC samples between previously described subgroups with respect to survival (better vs. poor prognosis) [21], cell origin (hepatocyte vs. hepatoblast) [22], activation of c‐MET/HGF (negative vs. positive) [20] and TGFβ [16] signaling (early vs. late) is indicated on the left side. (B) Statistical analysis (chi‐squared test) of HCC distribution between clusters 1 and 2 based on previous gene expression signatures and clinical parameters. (C) Kaplan–Meier plot and log‐rank statistics revealed a significant (*P* < 0.001) improved overall survival for patients included in cluster 1 (galunisertib‐associated). (D) Integrative transcriptomic analysis using galunisertib‐ and TGFβ‐associated signatures established in the PLC/PRF/5 cell line as performed in (A). Contrary to the SNU‐449 cell line, cluster 1 included untreated control PLC/PRF/5 cells and cells treated with TGFβ while cluster 2 included cells treated with galunisertib (in presence and absence of TGFβ). (E) Statistical analysis (chi‐squared test) of HCC distribution between clusters 1 and 2 based on previous gene expression signatures and clinical parameters. (F) Kaplan–Meier plot and log‐rank statistics revealed a significant (*P* < 0.01) decreased overall survival for patients included in cluster 2 (galunisertib‐associated).

**Fig. 4:**
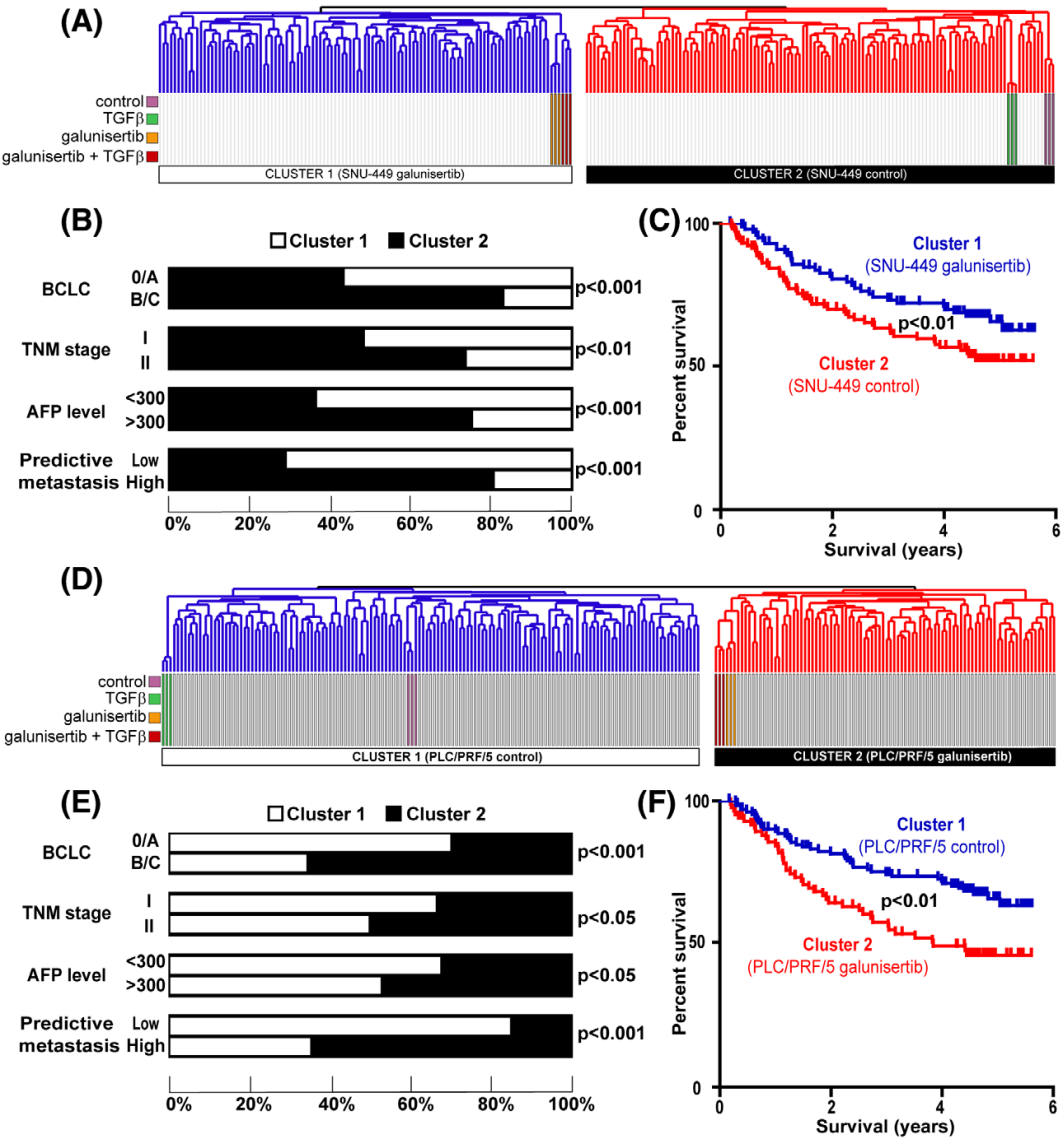
Validation of the clinical relevance of galunisertib‐ and TGFβ‐associated signatures derived from SNU‐449 and PLC/PRF/5 cells in an independent cohort of patients with HCC. (A) Dendrogram overview of *in vitro* experiments in SNU‐449 cells integrated with 225 cases of human HCC (NCI validating set, GSE14520). Clustering analysis was based on the expression of genes differentially expressed between the four conditions (i.e., SNU‐449 cells treated with TGFβ and galunisertib, alone or in combination, versus untreated control cells). Two main clusters were identified. Cluster 1 included cells treated with galunisertib (in presence and absence of TGFβ co‐treatment) and cluster 2 included untreated control cells and cells treated with TGFβ. (B) Statistical analysis (chi‐squared test) of HCC distribution between clusters 1 and 2 based on previous gene expression signatures and clinical parameters. Cluster 2 shows a significant enrichment in human HCC with the following features: advanced stage (BCLC B/C), TNM stage II, higher serum AFP level and a high risk of metastasis. (C) Kaplan–Meier plot and log‐rank statistics analysis revealed a significant decrease in overall survival for patients included in cluster 2 (*P* < 0.001). (D) Integrative transcriptomic analysis using galunisertib‐ and TGFβ‐associated signatures established in PLC/PRF/5 cells as performed in (A). Contrary to the SNU‐449 cell line, cluster 1 included untreated control PLC/PRF/5 cells and cells treated with TGFβ while cluster 2 included cells treated with galunisertib (in presence and absence of TGFβ). (E) Statistical analysis (chi‐squared test) of HCC distribution between clusters 1 and 2 based on previous gene expression signatures and clinical parameters. Cluster 2 shows a significant enrichment in human HCC with the following features: advanced stage (BCLC B/C), TNM stage II, higher serum AFP level and a high risk of metastasis. (F) Kaplan–Meier plot and log‐rank statistics analysis revealed a significant decrease in overall survival for patients included in cluster 2 (*P* < 0.001).

Gene set enrichment analysis suggested that whereas possibly beneficial in SNU‐449‐like HCC, galunisertib‐induced cell reprogramming could be detrimental in PLC/PRF/5‐like HCC, as regards limiting tumor progression and improving patient survival. To test this hypothesis from a clinical perspective, we integrated cell‐specific galunisertib and TGFβ signatures with the gene expression profiles of 139 cases of human HCC, as performed above. Accordingly, we first established and then integrated the gene expression profiles of PLC/PRF/5 and SNU‐449 cells treated with TGFβ in presence/absence of galunisertib (Fig. 3). For both cell lines, clustering analysis identified two clusters, one including the samples corresponding to untreated control cells or cells treated with TGFβ (thereafter referred to control) and another one including the samples corresponding to cells treated with galunisertib, alone or in combination with TGFβ (thereafter referred to galunisertib) (Fig. 3A,D). Interestingly, human HCC were not randomly distributed between the two clusters and the distribution appeared to be highly dependent on the cell line. Thus, in SNU‐449, the galunisertib cluster was associated with human HCC previously associated with a better prognosis (Fig. 3A), while the control cluster was associated with a poor prognosis (Fig. 3A) [21, 22]. Statistical analysis of HCC subgroups confirmed these observations since the galunisertib cluster included significantly more HCC associated with a good survival, no sign of vascular invasion and previously reported signatures of better prognosis, that is, the so‐called hepatocyte [22], c‐MET/HGF‐negative [20], and early TGFβ signatures [16] (Fig. 3B). Accordingly, human HCC from the galunisertib cluster exhibited a longer overall survival as compared to HCC from the control cluster (Fig. 3C). Suggesting that galunisertib could be effective in Hoshida's S1 HCC subtype, a phase 2 clinical trial demonstrated efficacy in TGFβ activated tumors with low alpha‐fetoprotein (AFP) level [13]. Similar to previous reports, galunisertib exerted limited antiproliferative effects in HCC cell lines [9, 37]. However, it was suggested that galunisertib exerted anti‐invasive properties in cell lines defined by the “late‐TGFβ signature” (similar to SNU‐449), in agreement with our results showing that the galunisertib signature derived from SNU‐449 cells was associated with better clinical outcomes. Very interestingly, performing the same analysis using the signatures established in PLC/PRF/5 cells resulted in completely opposite observations (Fig. 3D,E). Indeed, while the control and TGFβ‐associated clusters were recapitulating human HCC with a better prognosis, the galunisertib cluster recapitulated human HCC with poor prognosis signatures and a reduced overall survival (Fig. 3E,F). GSEA also highlighted that a statistically significant relationship between the HOSHIDA_LIVER_CANCER SUBCLASS_S1 subtype and human HCC with poor prognosis associated with the SNU‐449 signature. Conversely, a significant relationship between the HOSHIDA_LIVER_CANCER SUBCLASS_S3 subtype and human HCC with better prognosis associated with the PLC/PRF/5 signature was observed (Fig. S4A). More interestingly and fully supporting our results, the same approach using human HCC tumors classified according to the galunisertib signatures established in PLC/PRF/5 and SNU‐449 cell lines resulted in opposite observations (Fig. S4B,C). We further validated these results using an independent cohort of 225 human HCC (GSE14520) (Fig. 4). For the SNU‐449 cell line, data integration similarly identified two clusters, the galunisertib cluster being representative of HCC with a better prognosis as regard to the Barcelona Clinic Liver Cancer (BCLC) classification, Tumor Node Metastasis (TNM) staging, serum AFP level and metastasis propensity (Fig. 4A,B). Kaplan–Meier plot and log‐rank statistics confirmed that HCC in the galunisertib cluster exhibited a significantly better overall survival (Fig. 4C). On the contrary, for the PLC/PRF/5 cell line, the galunisertib cluster showed a significant enrichment in HCC associated with advanced BCLC and TNM stages (BCLC C and TNM stage III), a high serum AFP level (> 300 ng·mL^−1^), and a higher risk of metastasis (Fig. 4D,E). Accordingly, Kaplan–Meier plot and log‐rank statistics highlighted a significant decrease in overall survival for patients included in the galunisertib cluster (Fig. 4F). Collectively, these results demonstrated that galunisertib‐ and TGFβ‐induced gene expression profiles in PLC/PRF/5 and SNU‐449 cell lines recapitulated human HCC with different prognosis. More importantly, the results strongly suggest that galunisertib‐driven transcriptional reprogramming in PLC/PRF/5‐like HCC (i.e., possibly well‐differentiated early‐stage HCC) could be detrimental. However, a recent quantitative proteomic analysis highlighted a molecular heterogeneity of early‐stage HCC (BCLC 0 and A) and demonstrated an activation of the TGFβ pathway in HCC with microscopic vascular invasion, elevated AFP, shorter survival, and tumor recurrence [38]. Poor prognosis signatures were enriched in this early HCC subtype, including the late TGFβ signature [16]. Altogether, these data suggest that some early‐stage HCC could also be eligible for therapeutic inhibition of the TGFβ pathway.

### SERPINF2 as a putative biomarker for galunisertib in HCC

**Fig. 5:**
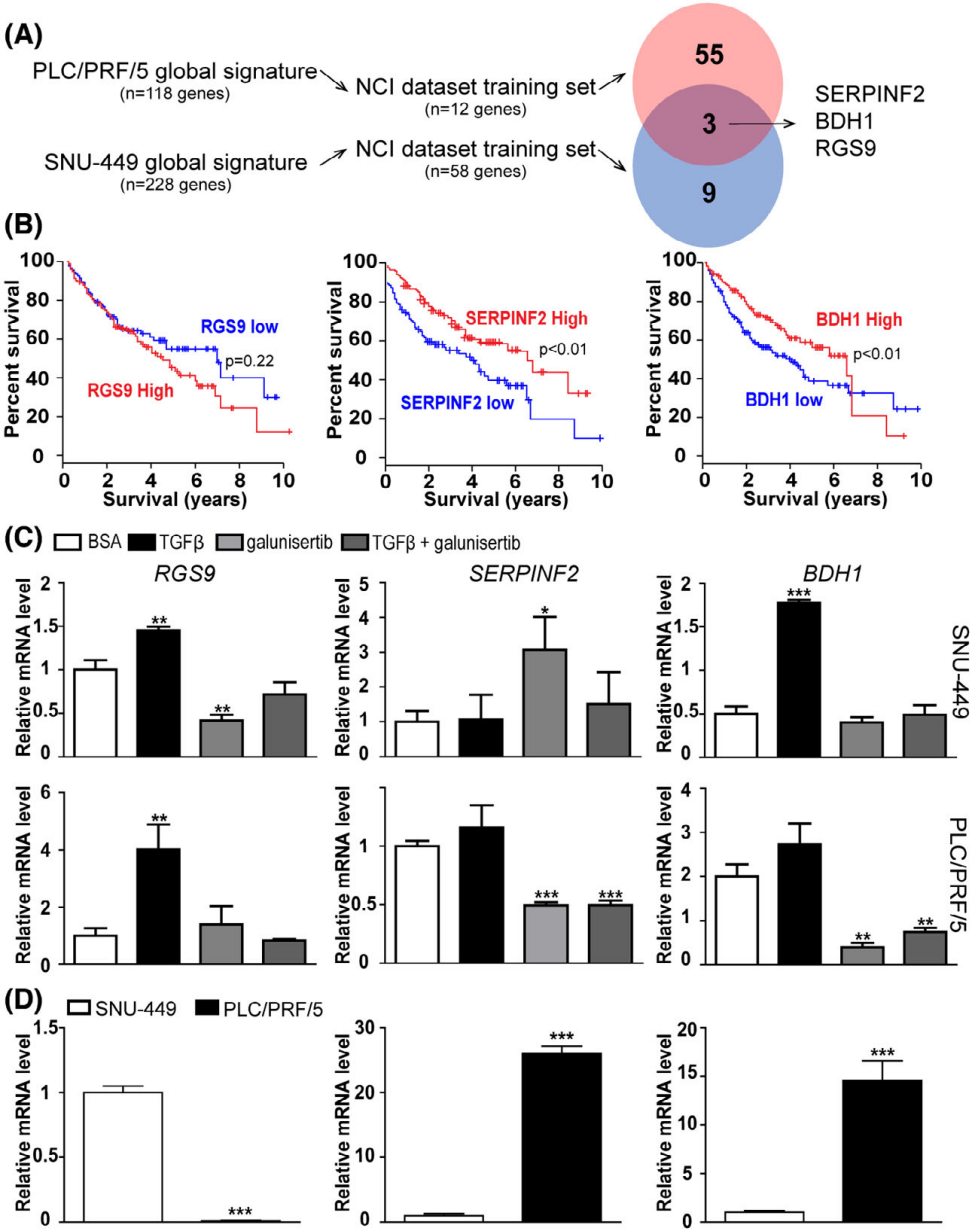
Identification of SERPINF2 as a putative marker of galunisertib efficacy in HCC. (A) Genes associated with the overall survival in human HCC from the galunisertib‐ and TGFβ‐associated signatures established in SNU‐449 and PLC/PRF/5 cells, as described in Fig. 3. Venn diagram unraveled three common genes negatively correlated with patient outcome. (B) Analysis of overall survival by Kaplan–Meier plot and log‐rank statistics in HCC from TCGA (LIHC dataset) based on the expression of candidate genes (blue curve: low expression; red curve: high expression). (C) Gene expression analysis of *RGS9*, *SERPINF2* and *BDH1* in SNU‐449 (upper panels) and PLC/PRF/5 (lower panels) cells treated with TGFβ and galunisertib, alone or in combination. (D) Gene expression analysis of *RGS9*, *SERPINF2* and *BDH1*, differentially expressed between SNU‐449 and PLC‐PRF/5 cells. In (C) and (D) data are presented as bar plots with mean ± SD and were compared using the Student's *t*‐test, *P < 0.05, **P < 0.01, and ***P < 0.001 (*n* = 3 independent experiments).

Identifying the patients who may benefit from galunisertib treatment represents a critical issue in the design of clinical trials given that inhibiting the TGFβ pathway in HCC in which TGFβ still limits tumor growth may contribute to tumor progression. However, very few biomarkers have been identified to distinguish those patients that may benefit from galunisertib from those that may not [30, 39]. A recent study suggests that SKIL, PMEPA1, and TGFB1 could be important biomarkers for selecting patients more likely to respond to galunisertib in HCC, although the mRNA levels of these biomarkers do not correlate with the levels of circulating proteins in patients [39]. Thus, from the galunisertib‐ and TGFβ‐associated signatures established in SNU‐449 and PLC/PRF/5 cell lines, we selected the genes significantly associated with patient survival (GSE1898 and GSE4024). From the 58 and 12 genes predictive of survival in SNU‐449 and PLC/PRF/5 signatures, respectively, only three genes were in common: *BDH1*, *RGS9*, and *SERPINF2* (Fig. 5A). We further validated in an independent HCC dataset (TCGA‐LIHC) that a high expression of *SERPINF2* or *BDH1* was significantly correlated with a better survival (Fig. 5B). Interestingly, the expression of *SERPINF2* was repressed by galunisertib in PLC/PRF/5 and induced in SNU‐449 cell line (Fig. 5C). At a basal level, a higher expression of *SERPINF2* was observed in PLC/PRF/5 cells (Fig. 5D). In addition, the expression of *SERPINF2* was able to define human HCC with variable prognosis, a high expression being associated with a better survival, no sign of vascular invasion, a well‐differentiated hepatocyte signature, a lower AFP level, and an early TGFβ signature (Fig. S5). Because *SERPINF2* encodes a secreted plasma protein, we suggest that it may serve as a clinically relevant companion biomarker for galunisertib in HCC. *SERPINF2* was previously identified among 41 hub driver‐genes predictive of differentiation, being expressed in well‐differentiated cell lines [29]. Up‐regulation of SERPINF2 in exosomes of good responders was also reported in rectal cancer patients with different responses to neoadjuvant radiotherapy [40]. In addition, SERPINF2 has been proposed as a serum marker of fibrosis progression for the follow‐up of patients hepatitis C [41] as well as an independent prognostic marker highly expressed in HCC with a better survival [42].

In conclusion, our study reports specific gene expression signatures suggestive of beneficial or adverse effects of galunisertib in HCC cell lines with clinical relevance in human HCC. How galunisertib drives detrimental effects in specific subsets of HCC remains to be elucidated. However, gene expression profiling presented in the study highlighted opposite pictures of galunisertib treatment in PLC/PRF/5 and SNU‐449 cell lines and suggested that galunisertib may favor an EMT‐like phenotype in well‐differentiated tumors by possibly repressing liver‐enriched transcription factors. It is also important to emphasize that our study only focused on the impact of the TGFβ pathway and its inhibition on the phenotype of tumor cells. However, TGFβ exerts critical impact on cells from the tumor microenvironment, including fibroblast‐like cells contributing to ECM remodeling and immune cells [6, 43, 44]. TGFβ contributes to the bidirectional cross‐talk between tumor cells and their microenvironment and thus to the regulation of tumor progression and response to therapies. Thus, it would be important to take into account this parameter in future studies [6].

## Conflict of interest

The authors declare no conflict of interest.

### Peer review

The peer review history for this article is available at https://www.webofscience.com/api/gateway/wos/peer‐review/10.1002/2211‐5463.13647.

## Author contributions

CC conceived and designed the project; MD, BM, KBe, GA, CL, and CC acquired the data; MD and CC analyzed and interpreted the data; MD and CC wrote the paper; LS, KBo, and CC acquired funding and supervised the study; CC, TF, and RL revised the manuscript; all authors reviewed and edited the manuscript.

## Supporting information


**Fig. S1.** SNU‐449 and PLC/PRF/5 cell lines exhibit a mesenchymal‐ and epithelial‐like phenotype, respectively. (A) Immunofluorescence micrographs of epithelial (CDH1) and mesenchymal (Vimentin, VIM) markers in SNU‐449 and PLC/PRF/5 cells at basal level. Scale bar: 50μm. (B) Quantitative PCR analysis of *CDH1* and *VIM* expression. ***P < 0.001; n=3. (C) Quantitative analysis by ELISA of TGFβ protein expression in the supernatant of SNU‐449 and PLC‐PRF‐5 cells after 48 hours of culture. In (B) and (C) data are presented as bar plots with mean ± SD and were compared using the Student's t test, ***P < 0.001 (n = 3 independent experiments).
**Fig. S2.** Relative expression of genes encoding liver‐enriched transcription factors in PLC/PRF/5 (upper panels) and SNU‐449 (lower panels) HCC cell lines at basal level (CTRL) as well as upon TGFβ (TGFb) and galunisertib (LY) exposure, alone (LY) or in combination (TGFb+LY). Data are presented as bar plots with mean ± SD and were compared using the Student's t test, *P < 0.05 (n = 3 independent experiments).
**Fig. S3.** Gene set enrichment analysis (GSEA) of non‐SMAD PI3K (upper 4 panels) and NFKB (lower 4 panels) signaling pathway signatures in the gene expression profiles of PLC/PRF/5 (A) and SNU‐449 (B) HCC cell lines upon TGFβ exposure, alone (TGFβ) or in combination with galunisertib (TGFβ+galunisertib). The following curated signatures have been used: REACTOME_CONSTITUTIVE_SIGNALING_BY_ABERRANT_PI3K_IN_CANCER.v2023.1.Hs.grp and NFKAPPAB_01.v2023.1.Hs.grp (https://www.gsea‐msigdb.org/gsea/msigdb/cards/). NES, normalized enrichment score, as determined by GSEA.
**Fig. S4.** Gene set enrichment analysis (GSEA) of human HCC tumors (139 cases from the GSE1898 and GSE4024 datasets) defined by the signature of genes differentially expressed between SNU‐449 and PLC/PRF/5 cell lines at basal level (A), as described in Fig. 1E, or upon galunisertib exposure in SNU‐449 cell line (B), as described in Fig. 3C or in PLC/PRF/5 cell line (C), as described in Fig. 3F. GSEA was focused on HOSHIDA_LIVER_CANCER SUBCLASS_S1 (left panels) or _S3 (right panels) signatures.
**Fig. S5.** Clinical relevance of human HCC (n=139 cases, GSE1898 and GSE4024) defined by a high (n=70 cases) versus low (n=69 cases) expression of SERPINF2 (median SERPINF2 expression was used to defined high versus low expressing groups). Statistical analysis (chi‐squared test) of HCC distribution between high versus low SERPINF2 expressing groups was based on previous gene expression signatures and clinical parameters.

**Table S1.** List of genes differentially expressed between the PLC/PRF/5 and SNU‐449 cell lines (fold‐change FC > 2; P < 0.001).

**Table S2.** List of genes differentially expressed by galunisertib in SNU‐449 cells (fold‐change FC > 2; P < 0.01).

**Table S3.** List of genes differentially expressed by galunisertib in PLC/PRF/5 cells (fold‐change FC > 2; P < 0.01).

## Data Availability

The datasets used and produced in this study are available from the gene expression omnibus database with the following accession numbers: GSE211806, GSE1898, GSE4024, and GSE14520 (e.g., https://www.ncbi.nlm.nih.gov/geo/query/acc.cgi?acc=GSE211806).
