# Supplementary material for: Transcriptomic evidence for tumor‐specific beneficial or adverse effects of TGFβ pathway inhibition on the prognosis of patients with liver cancer
Source: FEBS Open Bio. 2023 May 31;13(7):1278–90. doi: 10.1002/2211-5463.13647 (PMC10315808; doi:10.1002/2211-5463.13647)
Supplement: Supplementary file 1 — Fig. S1. SNU‐449 and PLC/PRF/5 cell lines exhibit a mesenchymal‐ and epithelial‐like phenotype, respectively. (A) Immunofluorescence micrographs of epithelial (CDH1) and mesenchymal (Vimentin, VIM) markers in SNU‐449 and PLC/PRF/5 cells at basal level. Scale bar: 50μm. (B) Quantitative PCR analysis of CDH1 and VIM expression. ***P < 0.001; n=3. (C) Quantitative analysis by ELISA of TGFβ protein expression in the supernatant of SNU‐449 and PLC‐PRF‐5 cells after 48 hours of culture. In (B) and (C) data are presented as bar plots with mean ± SD and were compared using the Student's t test, ***P < 0.001 (n = 3 independent experiments). Fig. S2. Relative expression of genes encoding liver‐enriched transcription factors in PLC/PRF/5 (upper panels) and SNU‐449 (lower panels) HCC cell lines at basal level (CTRL) as well as upon TGFβ (TGFb) and galunisertib (LY) exposure, alone (LY) or in combination (TGFb+LY). Data are presented as bar plots with mean ± SD and were compared using the Student's t test, *P < 0.05 (n = 3 independent experiments). Fig. S3. Gene set enrichment analysis (GSEA) of non‐SMAD PI3K (upper 4 panels) and NFKB (lower 4 panels) signaling pathway signatures in the gene expression profiles of PLC/PRF/5 (A) and SNU‐449 (B) HCC cell lines upon TGFβ exposure, alone (TGFβ) or in combination with galunisertib (TGFβ+galunisertib). The following curated signatures have been used: REACTOME_CONSTITUTIVE_SIGNALING_BY_ABERRANT_PI3K_IN_CANCER.v2023.1.Hs.grp and NFKAPPAB_01.v2023.1.Hs.grp (https://www.gsea‐msigdb.org/gsea/msigdb/cards/). NES, normalized enrichment score, as determined by GSEA. Fig. S4. Gene set enrichment analysis (GSEA) of human HCC tumors (139 cases from the GSE1898 and GSE4024 datasets) defined by the signature of genes differentially expressed between SNU‐449 and PLC/PRF/5 cell lines at basal level (A), as described in Fig. 1E, or upon galunisertib exposure in SNU‐449 cell line (B), as described in Fig. 3C or in PLC/PRF/5 cell line (C), as described [file FEB4-13-1278-s001.docx]

**Supporting Information**

**
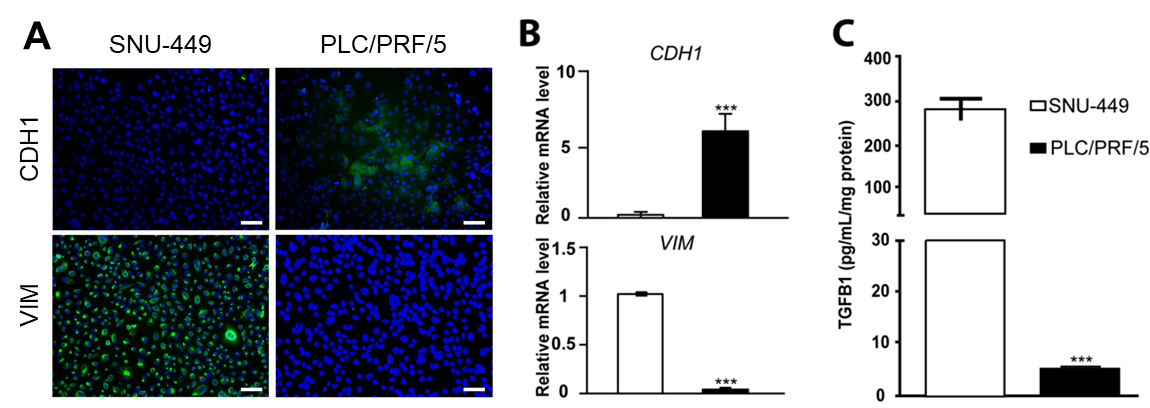
**

**Fig. S1**

SNU-449 and PLC/PRF/5 cell lines exhibit a mesenchymal- and epithelial-like phenotype, respectively. (A) Immunofluorescence micrographs of epithelial (CDH1) and mesenchymal (Vimentin, VIM) markers in SNU-449 and PLC/PRF/5 cells at basal level. Scale bar: 50µm. (B) Quantitative PCR analysis of *CDH1* and *VIM* expression. ***P < 0.001; n=3. (C) Quantitative analysis by ELISA of TGFβ protein expression in the supernatant of SNU-449 and PLC-PRF-5 cells after 48 hours of culture. In (B) and (C) data are presented as bar plots with mean ± SD and were compared using the Student's t test, ***P < 0.001 (n = 3 independent experiments).


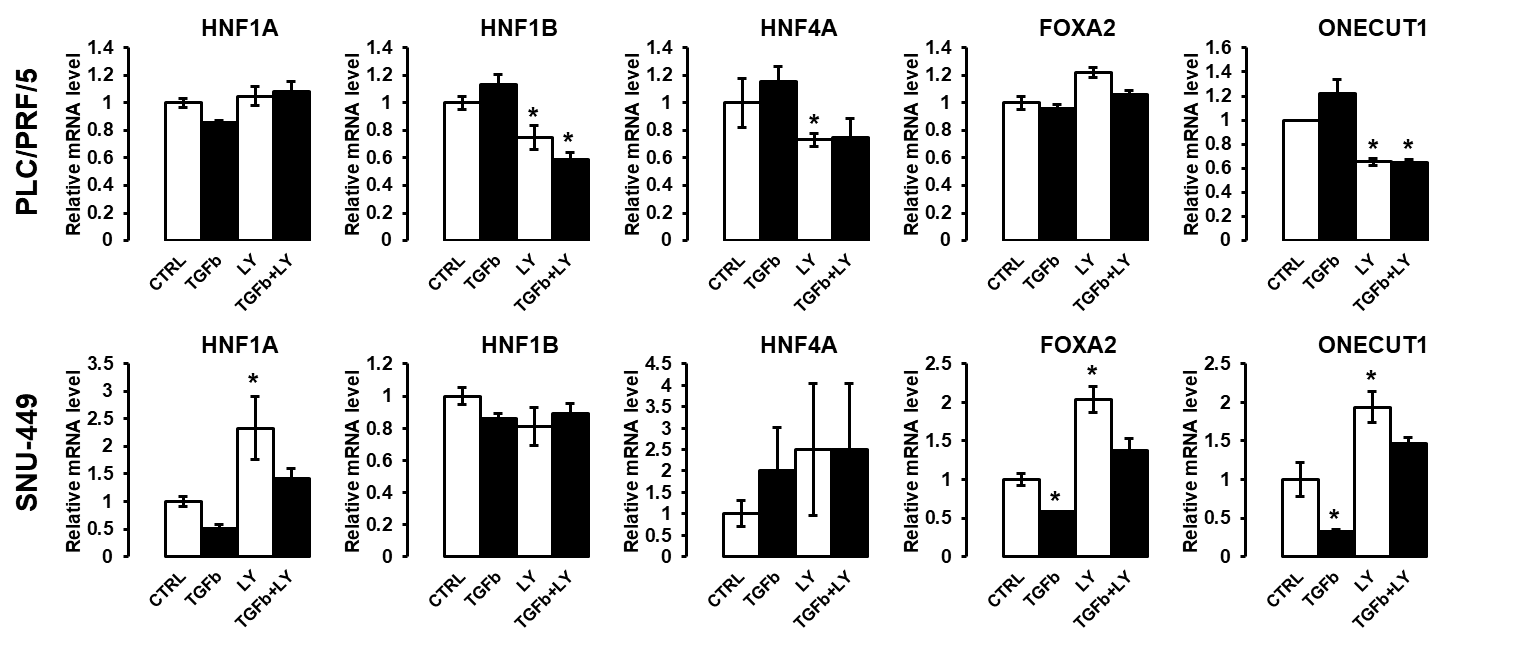


**Fig. S2**

Relative expression of genes encoding liver-enriched transcription factors in PLC/PRF/5 (upper panels) and SNU-449 (lower panels) HCC cell lines at basal level (CTRL) as well as upon TGFβ (TGFb) and galunisertib (LY) exposure, alone (LY) or in combination (TGFb+LY). Data are presented as bar plots with mean ± SD and were compared using the Student's t test, *P < 0.05 (n = 3 independent experiments).


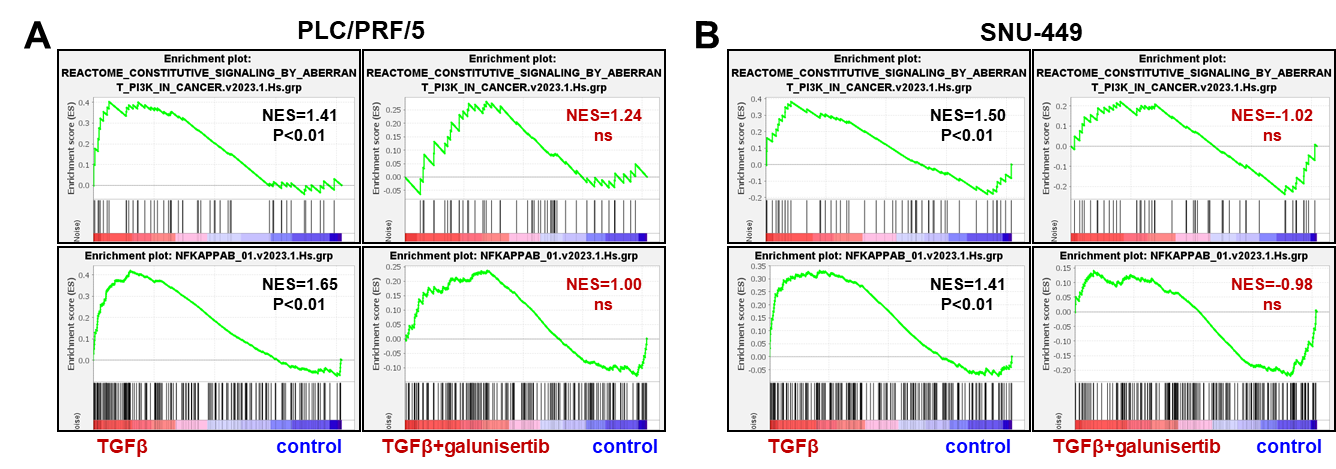


**Fig. S3**

Gene set enrichment analysis (GSEA) of non-SMAD PI3K (upper 4 panels) and NFKB (lower 4 panels) signaling pathway signatures in the gene expression profiles of PLC/PRF/5 (A) and SNU-449 (B) HCC cell lines upon TGFβ exposure, alone (TGFβ) or in combination with galunisertib (TGFβ+galunisertib). The following curated signatures have been used: REACTOME_CONSTITUTIVE_SIGNALING_BY_ABERRANT_PI3K_IN_CANCER.v2023.1.Hs.grp and NFKAPPAB_01.v2023.1.Hs.grp (<https://www.gsea-msigdb.org/gsea/msigdb/cards/>). NES, normalized enrichment score, as determined by GSEA.


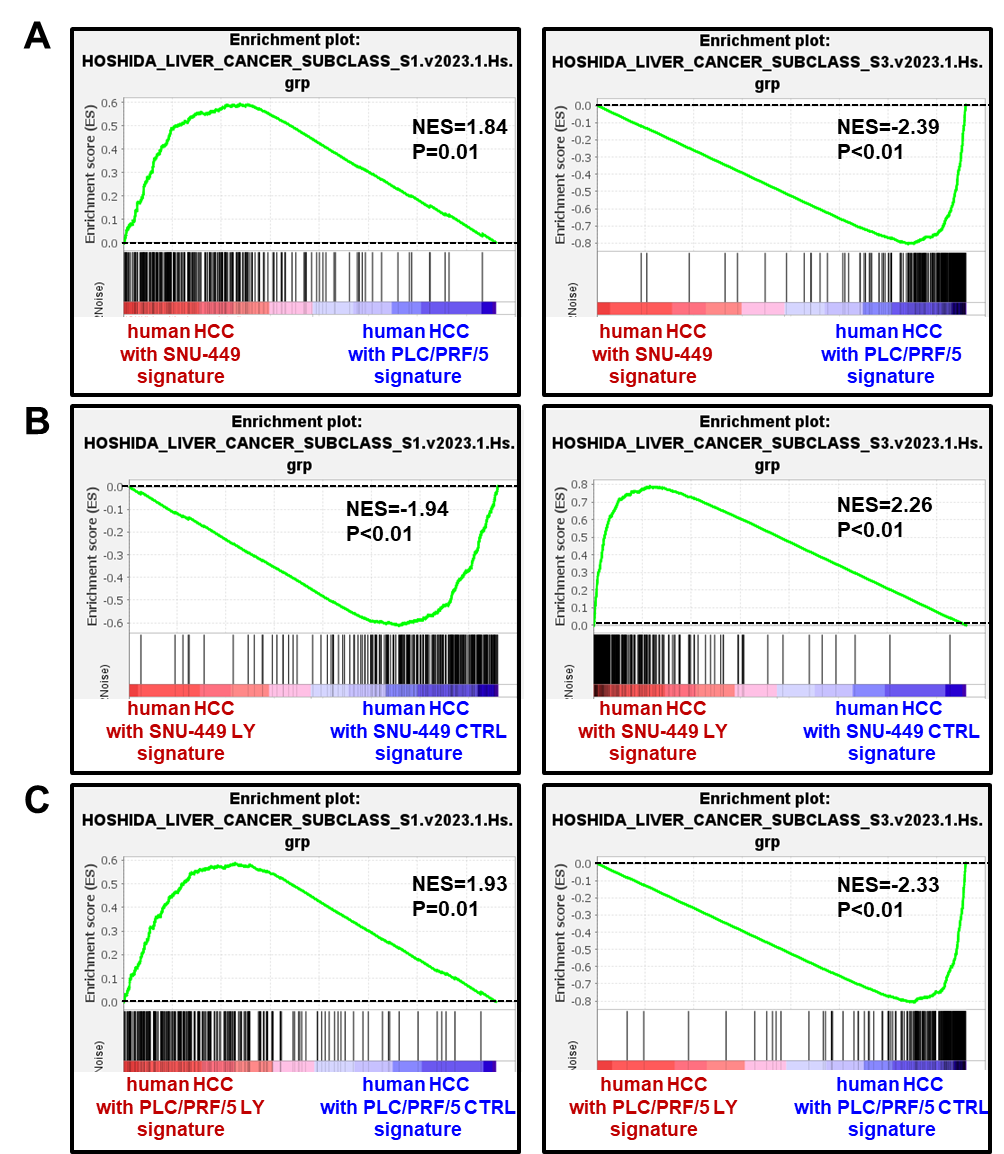


**Fig. S4**

Gene set enrichment analysis (GSEA) of human HCC tumors (139 cases from the GSE1898 and GSE4024 datasets) defined by the signature of genes differentially expressed between SNU-449 and PLC/PRF/5 cell lines at basal level (A), as described in Fig. 1E, or upon galunisertib exposure in SNU-449 cell line (B), as described in Fig. 3C or in PLC/PRF/5 cell line (C), as described in Fig. 3F. GSEA was focused on HOSHIDA_LIVER_CANCER SUBCLASS_S1 (left panels) or _S3 (right panels) signatures.


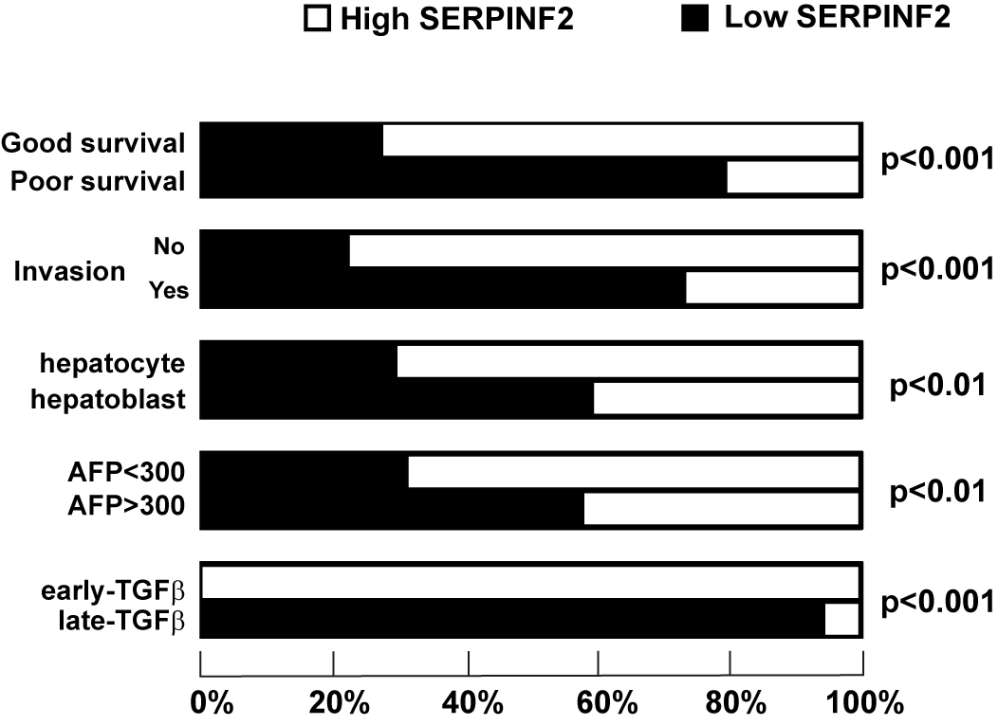


**Fig. S5**

Clinical relevance of human HCC (n=139 cases, GSE1898 and GSE4024) defined by a high (n=70 cases) versus low (n=69 cases) expression of SERPINF2 (median SERPINF2 expression was used to defined high versus low expressing groups). Statistical analysis (chi-squared test) of HCC distribution between high versus low SERPINF2 expressing groups was based on previous gene expression signatures and clinical parameters.

**Supplementary Tables**

Tables are provided as separate .xls files

**Table S1.** List of genes differentially expressed between the PLC/PRF/5 and SNU-449 cell lines (fold-change FC>2; P < 0.001)

**Table S2.** List of genes differentially expressed by galunisertib in SNU-449 cells (fold-change FC>2; P < 0.01)

**Table S3.** List of genes differentially expressed by galunisertib in PLC/PRF/5 cells (fold-change FC>2; P < 0.01)
